# Effects of reduced carbonic anhydrase activity on CO_2_ assimilation rates in *Setaria viridis*: a transgenic analysis

**DOI:** 10.1093/jxb/erw357

**Published:** 2016-10-04

**Authors:** Hannah L Osborn, Hugo Alonso-Cantabrana, Robert E Sharwood, Sarah Covshoff, John R Evans, Robert T Furbank, Susanne von Caemmerer

**Affiliations:** 1 Australian Research Council Centre of Excellence for Translational Photosynthesis, Division of Plant Sciences, Research School of Biology, The Australian National University, Acton, ACT, Australia; 2 Department of Plant Sciences, University of Cambridge, Cambridge, UK

**Keywords:** Carbonic anhydrase, C^18^O^16^O isotope discrimination, C_4_ photosynthesis, mesophyll conductance, *Setaria viridis*, transformation

## Abstract

In C_4_ species, the major β-carbonic anhydrase (β-CA) localized in the mesophyll cytosol catalyses the hydration of CO_2_ to HCO_3_^−^, which phospho*enol*pyruvate carboxylase uses in the first step of C_4_ photosynthesis. To address the role of CA in C_4_ photosynthesis, we generated transgenic *Setaria viridis* depleted in β-CA. Independent lines were identified with as little as 13% of wild-type CA. No photosynthetic defect was observed in the transformed lines at ambient CO_2_ partial pressure (*p*CO_2_). At low *p*CO_2_, a strong correlation between CO_2_ assimilation rates and CA hydration rates was observed. C^18^O^16^O isotope discrimination was used to estimate the mesophyll conductance to CO_2_ diffusion from the intercellular air space to the mesophyll cytosol (*g*_m_) in control plants, which allowed us to calculate CA activities in the mesophyll cytosol (*C*_m_). This revealed a strong relationship between the initial slope of the response of the CO_2_ assimilation rate to cytosolic *p*CO_2_ (*AC*_m_) and cytosolic CA activity. However, the relationship between the initial slope of the response of CO_2_ assimilation to intercellular *p*CO_2_ (*AC*_i_) and cytosolic CA activity was curvilinear. This indicated that in *S. viridis*, mesophyll conductance may be a contributing limiting factor alongside CA activity to CO_2_ assimilation rates at low *p*CO_2_.

## Teaser

Carbonic anhydrase and mesophyll conductance are both limiting factors affecting CO_2_ assimilation rates at low *p*CO_2_ as examined in stably transformed lines of the C_4_ species, *Setaria viridis*.

## Introduction

C_4_ plants have evolved a CO_2_-concentrating mechanism (CCM) that enables the elevation of CO_2_ around the active sites of Rubisco by a combination of anatomical and biochemical specialization (Hatch, 1987). C_4_ photosynthesis has independently evolved >60 times, providing one of the most widespread and effective solutions for remedying the catalytic inefficiency of Rubisco (Sage *et al.*, 2012; Christin and Osborne, 2013). The key carboxylases in C_4_ plants are localized to different cellular compartments. Phospho*enol*pyruvate carboxylase (PEPC) is localized to the cytosol of mesophyll cells and Rubisco to the chloroplasts of bundle sheath cells. For the CCM to operate effectively, PEPC activity must exceed Rubisco activity to balance leakage of CO_2_ out of the bundle sheath compartment. This maintains a high bundle sheath CO_2_ level but prevents wasteful overcycling of the mesophyll CO_2_ ‘pump’ (von Caemmerer and Furbank, 2003). As PEPC utilizes HCO_3_^−^ and not CO_2_, the first committed enzyme of the C_4_ pathway is carbonic anhydrase (CA) which catalyses the reversible conversion of CO_2_ and HCO_3_^−^ in the cytosol of mesophyll cells. C_4_ acids produced by PEPC then diffuse into the bundle sheath cells where they are decarboxylated, supplying CO_2_ for Rubisco.

Within higher plants, there are multiple forms of the α-CA, β-CA, and γ-CA families which share little sequence homology (Moroney *et al.*, 2001). β-CAs are the most prevalent CA family in land plants. CA is an abundant enzyme in C_3_ plants, representing up to 2% of the soluble leaf protein (Okabe *et al.*, 1984). In C_3_ plants, the role of CA is unclear (Badger and Price, 1994) as it does not appear to limit photosynthesis but does influence stomatal conductance, guard cell movement, and amino acid biosynthesis (Hu *et al.*, 2010; DiMario *et al.*, 2016; Engineer *et al.*, 2016).

It has long been contended that the uncatalysed rate of CO_2_ conversion to HCO_3_^−^ is insufficient to support C_4_ photosynthetic flux (Hatch and Burnell, 1990; Badger and Price, 1994). This hypothesis was supported by experiments in the C_4_ dicot *Flaveria bidentis*, where antisense plants with <10% of wild-type CA activity required high CO_2_ for growth and showed reduced CO_2_ assimilation rates (von Caemmerer *et al.*, 2004; Cousins *et al.*, 2006). However, in the C_4_ monocot *Zea mays* mutant plants with reduced CA activity (3% of wild type), no limitation to CO_2_ assimilation rates at ambient CO_2_ was observed (Studer *et al.*, 2014). CA activity has been shown to vary widely between species (Cousins *et al.*, 2008), and it is unclear whether CA activities are limiting at high CO_2_ assimilation rates, as has previously been suggested (Hatch and Burnell, 1990; Gillon and Yakir, 2000).

We examined the role of CA in the model C_4_ monocot species *Setaria viridis* (green foxtail millet). *Setaria viridis* is closely related to agronomically important C_4_ crops including *Z. mays* (maize), *Sorghum bicolor* (sorghum), and *Saccharum officinarum* (sugarcane) (Brutnell *et al.*, 2010). It is an ideal model species due to its rapid generation time, small stature, high seed production, diploid status, and small genome that is sequenced and publicly available (Doust *et al.*, 2009; Brutnell *et al.*, 2010; Li and Brutnell, 2011). Here we used a stable transformation approach to examine the role of CA in *S. viridis* and could show that *S. viridis* is a useful model species that lends itself to molecular manipulation of the C_4_ photosynthetic pathway. Two constructs both targeting the major leaf β-CA (Si003882m.g) were used to generate three independent transformed lines with reduced CA activity. A strong correlation between the CO_2_ assimilation rate at low *p*CO_2_ and CA activity was observed. Our combined measurements of mesophyll conductance and CA activity suggest that increasing mesophyll conductance may be an important way to increase the CO_2_ assimilation rate at low intercellular *p*CO_2_, as may occur under drought.

## Materials and methods

### Plant growth conditions

T_1_ seeds were incubated in 5% liquid smoke (Wrights) for 24h to promote germination, and germinated in garden soil mix fertilized with Osmocote (Scotts, Australia) in small containers before being transferred to individual 2 litre pots. Plants were grown in controlled environmental chambers, irradiance 500 µmol photons m^−2^ s^−1^, 16h photoperiod, 28 °C day, 24 °C night, 2% CO_2_. Pots were watered daily.

### Construct generation

Two different constructs were used to generate three lines of reduced CA activity. First, an RNAi was targeted to the primary leaf β-CA Si003882m which generated lines 2.1 and 5.3. A region of Si003882m.g was amplified by PCR using gene-specific primers (Supplementary Table S1 at *JXB* online) and reverse-transcribed RNA from *S. viridis* leaves ligated into pENTR/D-TOPO (ThermoFisher), and verified by sequencing. The fragment was inserted via a double Gateway system LR reaction (Invitrogen) into the hairpin RNAi binary vector pSTARGATE (Greenup *et al.*, 2010) to form a stem–loop region under the control of the ubiquitin promoter/intron (UBI) and octopine synthase (OCS) terminator to form the RNAi vector pSG/*CAa*.

Secondly, an overexpression approach, which resulted in gene silencing, generated the third transformed line, 1.1. The coding sequence of the maize β-CA gene (GRMZM2G348512), *ZmCA2* (Studer *et al.*, 2014), was amplified by reverse transcription–PCR (RT–PCR) from total RNA extracted from B73 maize. Total RNA was isolated using hot acid phenol and chloroform, and then treated with RQ1 RNase-free DNase (Promega). The reverse transcription and PCRs were performed as per the manufacturer’s protocols with Superscript II (ThermoFisher) and Phusion High-Fidelity DNA polymerase (NEB), respectively (for primers, see Supplementary Table S1). The sequence encoding an AcV5 epitope tag (Lawrence *et al.*, 2003) was added to the C-terminal end of *ZmCA2*. The resulting *ZmCA2* amplicon was cloned into pENTR/D-TOPO and verified by sequencing. LR Gateway cloning (ThermoFisher) was used to insert the *ZmCA2* coding sequence into the overexpression vector, pSC110. pSC110 was created by Gibson Assembly (Gibson *et al.*, 2009) from two modified pMDC164 vectors (Curtis and Grossniklaus, 2003), kindly provided to us by Udo Gowik (Heinrich-Heine University, Dusseldorf, Germany). *ZmCA2* expression from pSC110 was driven by the B73 *ZmPEPC* promoter. pSC110 and pSC110/*ZmCA2* were verified by sequencing.

Both constructs were transformed into *Agrobacterium tumefaciens* strain AGL1 for stable plant transformation.

### Callus induction and plant transformation

Stable transformation of *S. viridis* (accession A10.1) was carried out as described by Brutnell *et al.* (2010). Seed coats were mechanically removed from mature *S. viridis* seeds to improve germination. Seeds were sterilized before plating on callus induction medium [CIM; 4.3g l^−1^ Murashige and Skoog (MS) salts, pH 5.8, 10ml l^−1^ 100× MS vitamins stock, 40g l^−1^ maltose, 35mg l^−1^ ZnSO_4_·7H_2_O, 0.6mg l^−1^ CuSO_4_·5H_2_O, 4g l^−1^ Gelzan, 0.5mg l^−1^ kinetin, 2mg l^−1^ 2,4-D]. After 4 weeks in the dark at 24 °C, any seedling structures or gelatinous calli were removed and remaining calli transferred to fresh CIM. After a further 2 weeks, calli were divided and replated onto fresh CIM. One week later, transformations were performed.

AGL1 containing the construct of interest were grown in the presence of 50 µg l^−1^ kanamycin and 50 µg l^−1^ rifampicin at 28 °C to OD_600_=0.5 and then resuspended in CIM without Gelzan and hormones. Acetosyringone (200mM) and synperonic [0.01% (w/v)] were added to the *Agrobacterium* solution before incubating the calli in the medium for 5min at room temperature. The calli were blotted dry on sterile filter paper and incubated at 22 °C for 3 d in the dark. The calli were then transferred to selective CIM (CIM containing 40mg l^−1^ hygromycin, 150mg l^−1^ timentin) and incubated in the dark at 24 °C for 16 d. Calli were then transferred to selective plant regeneration medium (PRM) containing 4.3g l^−1^ MS salts, pH 5.8, 10ml l^−1^ 100× MS vitamins, 20g l^−1^ sucrose, 7g l^−1^ Phytoblend, 2mg l^−1^ kinetin, 150mg l^−1^ timentin, 15mg l^−1^ hygromycin. Calli were maintained at 24 °C under a 16h light:8h dark photoperiod and a light intensity of 60 µmol photons m^−2^ s^−1^. Developing shoots were transferred to selective rooting medium (RM) containing 2.15g l^−1^ MS salts, pH 5.7, 10ml l^−1^ 100× MS vitamins, 30g l^−1^ sucrose, 7g l^−1^ Phytoblend, 150mg l^−1^ timentin, 20mg l^−1^ hygromycin. Shoots that survived and developed roots were genotyped using primers against the hygromycin phosphotransferase gene (Supplementary Table S1) by PCR, and positive transformants were transplanted to soil.

### Selection of plants for analysis

The progeny of three independent T_0_ transformation events were analysed for CA hydration rates (Supplementary Fig. S1). One T_1_ plant with low CA hydration rates was selected from each transformation event (labelled 5.3, 2.1, and 1.1) and its progeny (T_2_) used for all future analysis. Two sets of experiments were performed on the T_2_ plants. First, gas exchange and biochemical analysis on lines 5.3, 2.1, and 1.1 (Table 1) and, secondly, gas exchange and oxygen discrimination on lines 5.3 and 1.1 (Table 2). Each T_2_ plant was genotyped prior to experiments using primers against the hygromycin phosphotransferase gene (Supplementary Table S1). The progeny of a plant which went through the *S. viridis* transformation process and tested negative for the hygromycin phosphotransferase gene were used as null controls.

**Table 1.** Physiological and biochemical characteristics of CA transformants under ambient CO_2_ conditions Net CO_2_ assimilation rate (*A*), stomatal conductance (*g*_s_), mesophyll *p*CO_2_ (*C*_m_), the rate constant of CA hydration (*k*_CA_), and enzyme activities were measured from the uppermost, fully expanded leaf of 5-week-old plants grown at 2% CO_2_. Gas exchange measurements were made at 25 °C leaf temperature, flow rate at 500 µmol m^−2^ s^−1^, and irradiance of 1500 µmol photons m^−2^ s^−1^. Three T_2_ plants from three different transformation events were measured.

	*A*	*g*_s_	*C*_m_	*k*_CA_	Rubisco	PEPC	NADP-ME
µmol m^−2^ s^−1^	mol m^−2^ s^−1^	µbar	mol m^−2^ s^−1^ bar^−1^	µmol m^−2^ s^−1^	µmol m^−2^ s^−1^	µmol m^−2^ s^−1^	
**Null**	22.5±0.6 a	0.19±0.01 a	132.4±3.3 a	6.1±0.8 a	18.7±1.5 a	229.6±19.3 a	59.8±4.3 a
**5.3**	21.7±2.6 a	0.2±0.02 a	118.9±13.1 a	3.3±0.2 b	18.8±1.8 a	249.3±24.6 a	54.5±5.8 a
**2.1**	18.5±1.9 a	0.16±0.01 a	152.9±15.2 a	2.0±0.2 b,c	20.9±2.9 a	181.5±25.4 a	47.3±2.6 a
**1.1**	19.1±1.2 a	0.19±0.02 a	153.9±4.4 a	0.8±0.1 c	19.7±1.8 a	180.3±18.4 a	43.6±3.9 a

Significant differences are based on one-way ANOVA and Tukey post-hoc analysis (SPSS statistics version 22; *P*=0.05).

**Table 2.** Physiological characteristics of CA transformants at ambient CO_2_ measured using LI-6400XT coupled to a tunable diode laser Net CO_2_ assimilation rate (*A*), stomatal conductance (*g*_s_), mesophyll *p*CO_2_ (*C*_m_), the ratio of intercellular to ambient *p*CO_2_ (*C*_i_/*C*_a_), the rate constant of CA hydration (*k*_CA_), online Δ^18^O discrimination, and the length of mesophyll cells exposed to intercellular airspace (*S*_m_) were measured on the uppermost, fully expanded leaf of 5-week-old plants grown at 2% CO_2_. Gas exchange measurements were made at 2% O_2_, 25 °C leaf temperature, flow rate at 500 µmol m^−2^ s^−1^, and irradiance of 1500 µmol photons m^−2^ s^−1^. Three T_2_ plants from two different transformation events were measured.

	*A*	*g*_s_	*C*_m_	*C*_i_/*C*_a_	*k*_CA_	Δ^18^O	*S*_m_
µmol m^−2^ s^−1^	mol m^−2^ s^−1^	µbar	µbar	mol m^−2^ s^−1^ bar^−1^	‰	m^2^ m^−2^	
**Null**	30.0±1.4 a	0.30±0.03 a	144.6±5.9 a	0.39±0.03 a	8.4±0.7 a	18.0±1.4 a	10.2±0.4 a
**5.3**	29.2±0.9 a	0.29±0.02 a	157.9±10.5 a	0.34±0.01 a	2.5±0.3 b	13.6±0.7 a,b	–
**1.1**	24.5±1.6 a	0.26±0.03 a	178.1±13.5 a	0.43±0.02 a	0.8±0.2 b	10.9±0.6 b	10.2±0.9 a

Significant differences are based on one-way ANOVA and Tukey post-hoc analysis (SPSS statistics version 22; *P*=0.05).

### Insertion number estimation

DNA was isolated from a fully expanded leaf using a CTAB (cetyltrimethylammonium bromide) extraction buffer [2% CTAB (v/v), 20mM Tris–HCl pH 8, 1.4M NaCl, 20mM EDTA, 1% polyvinylpyrrolidone (PVP)-40 (w/v), 0.2% (v/v) β-mercaptoethanol] followed by extraction with phenol/chloroform/isoamylalcohol (25:24:1) and ethanol clean-up. DNA quality and quantity was determined using a NanoDrop spectrophotometer (Thermo Scientific).

IDNA genetics (UK) performed quantitative real-time PCR (qPCR) analysis to estimate the numbers of transgene copies in the CA transformed lines following the procedure described in Bartlett *et al.* (2008) with some modifications. The hygromycin phosphotransferase gene (with a FAM reporter) and the internal positive control (IPC, with a VIC reporter) were amplified together in a multiplex reaction (15min denaturation, then 40 cycles of 15s at 95 °C and 60s at 60 °C) in an ABI1900 real-time PCR machine. Fluorescence from the FAM and VIC fluorochromes was measured during each 60 °C step and the Ct values obtained. The difference between the Ct values for the hygromycin phosphotransferase gene and the IPC (the Delta Ct) was used to allocate the assayed samples into groups with the same gene copy number.

### RNA extraction and reverse transcription–quantitative PCR (RT–qPCR)

Leaf discs (0.78cm^2^) frozen and stored at −80 °C were lysed using the Qiagen TissueLyser II. RNA was extracted using the Trizol extraction method and in the presence of RNase inhibitor (Ambion). DNA was removed using the TURBO DNA free kit (Ambion), and RNA quantity and quality were determined using a NanoDrop (Thermo Scientific).

RNA (200ng) was reverse transcribed into cDNA using Qiagen’s RT^2^ HT First Strand cDNA synthesis kit. RT–qPCR and melt curve analysis were performed on a Viia7 Real-time PCR system using the Power SYBR green PCR Master Mix (Thermo Fisher) according to the manufacturer’s instructions. Primers (Supplementary Table S1) were designed using Primer3 in Geneious R7.1.6, ensuring products spanned an intron. Primer amplification efficiencies were determined by the Ct slope method; efficiencies for all primer pairs were comparable (~95%) and no amplification was detected in the no template control. Relative fold change was calculated by the ΔΔCt method, using the average of three nulls as reference, as described by Livak and Schmittgen (2001). The geometric mean of the Ct values for three reference genes was used for normalization (Vandesompele *et al.*, 2002). Statistics were performed with SigmaPlot (version 11.0).

### Determination of enzyme activities

For CA activity, leaf discs (0.78cm^2^) were collected from the uppermost fully expanded leaf of 5-week-old *S. viridis* plants and frozen in liquid nitrogen. Soluble protein was extracted by grinding one frozen leaf disc in ice-cold glass homogenizers (Tenbroek) in 500 µl of extraction buffer [50mM HEPES, pH 7.8, 1% (w/v) PVP, 1mM EDTA, 10mM dithiothreitol, 0.1% (v/v) Triton X-100, 2% (v/v) protease inhibitor cocktail (Sigma)]. Crude extracts were centrifuged at 4 °C for 1min at 13 000 *g* and the supernatant collected for the soluble CA assay. Activity was measured on a membrane inlet mass spectrometer to measure the rates of ^18^O exchange from labelled ^13^C^18^O_2_ to H_2_^16^O at 25 °C (Badger and Price, 1989; von Caemmerer *et al.*, 2004). The hydration rates were calculated as described by Jenkins *et al.* (1989).

For Rubisco, PEPC, and NADP-malic enzyme (ME) activities, soluble protein was extracted from fresh leaf discs collected from leaves used for gas exchange analysis. Spectrophotometric assays were then performed as described previously (Pengelly *et al.*, 2010, 2012; Sharwood *et al.*, 2016).

### Gas exchange measurements

Net photosynthesis (*A*) was measured over a range of intercellular *p*CO_2_ (*C*_i_) on the uppermost, fully expanded leaf of 5-week-old *S. viridis* plants using a portable gas exchange system LI-COR 6400XT (LI-COR Biosciences). Measurements were made after leaves had equilibrated at 380 µbar, flow rate 500 µmol s^−1^, leaf temperature 25 °C, and irradiance 1500 µmol photons m^−2^ s^−1^. CO_2_ response curves were measured in a stepwise increase (3min intervals) in CO_2_ partial pressure 380, 0, 23.75, 47.5, 71.25, 95, 142.5, 190, 285, 380, 570, 760, and 950 µbar whilst maintaining leaf temperature and irradiance conditions.

### Measurements of C^18^O^16^O discrimination (Δ^18^O)

Simultaneous measurements of exchange of CO_2_, H_2_O, C^18^O^16^O, and H_2_^18^O were made by coupling two LI-6400XT gas exchange systems to a tunable diode laser (TDL: TGA200A, Campbell Scientific Inc., Logan, UT, USA) to measure C^18^O^16^O and a Cavity Ring-Down Spectrometer (L2130-i, Picarro Inc., Sunnyvale, CA, USA) to measure the oxygen isotope composition of water vapour. The system is essentially that described by Tazoe *et al.* (2011) except that the TGA100 was replaced by a TGA200A and the additional laser for water vapour measurements has been added together with a 16 port distribution manifold. To generate gas flows to the gas exchange systems, N_2_ and O_2_ were mixed by mass flow controllers (Omega Engineering Inc., Stamford, CT, USA) to generate CO_2_-free air with 2% O_2_. The humidity of incoming air was adjusted by varying the temperature of water circulating around a Nafion tube (Permapure, MH-110-12P-4) but was kept constant in this set of experiments to supply water vapour of a constant ^18^O composition. To supply flow to the TDL and the L2130-i from the sample and reference gas streams, two T junctions were inserted into the match valve tubing and in the reference line of the LI-6400XT, respectively. This allowed leaves of two plants to be measured in sequence, with each LI-6400XT sampled by the TDL at 4min intervals for 20s at the sample and reference line. The Picarro Cavity Ring Down spectrometer sampled for 3min, so that leaves were sampled at 6min intervals.

Supplementary Fig. 5 shows the CO_2_ dependence of the standard error of δ^18^O of CO_2_ in the reference gas of repeated measurements on the TGA200A. The ^18^O isotopic composition of the CO_2_ calibration gas was 22.17±0.04‰ for Vienna mean oceanic water (VSMOW) and was checked against standards on an Isoprime mass spectrometer. We monitored the ^18^O composition of water vapour of the reference air streams daily, and the values were −6.07±0.08‰ and −6.34±0.08‰ (VSMOW) for LI-6400XT L1 and L2 references, respectively. We attribute the small difference between the reference lines to differences in the Nafion tubing. At the end of the experiment, the calibration of the Picarro L2130-i was confirmed by collecting water vapour samples from the gas stream of the LI-6400XT reference lines going to the Picarro as described by Cousins *et al.* (2006) and assaying these water samples against standards on a Picarro 1102i, which was set up to measure the ^18^O isotopic composition of water samples.

Gas exchange was measured on the uppermost fully expanded leaf of 5-week-old *S. viridis* plants at 25 °C, and leaves were equilibrated at ambient CO_2_ (380 µbar), irradiance 1500 µmol photons m^−2^ s^−1^, and 2% O_2_. The flow rate was 200 µmol s^−1^. CO_2_ concentration was adjusted from 380 to 760, 570, 380, and 190 µbar at 1h intervals. Immediately following gas exchange measurements, leaf discs were collected and stored at −80 °C until measurements of CA activity were made.

### Calculations of C^18^O^16^O (Δ^18^O) discrimination and mesophyll conductance (*g*_m_)

Discrimination against ^18^O in CO_2_ during photosynthesis, Δ^18^O, was calculated from the isotopic composition of the CO_2_ entering δ_in_ and exiting δ_out_ the leaf chamber and the CO_2_ concentration entering *C*_in_ and exiting *C*_out_ (all measured with the TDL) (Evans *et al.*, 1986; Barbour *et al.*, 2016;):

$$\Delta^{18}\text{O}=\frac{\xi \left(\delta_{out}-\delta_{in}\right)}{1+\delta_{out}-\xi \left(\delta_{out}-\delta_{in}\right)}$$ (1)

where ξ=*C*_in_/*C*_in_–*C*_out_. Sample streams were passed through a Nafion drying tube before entering the TDL, and CO_2_ values presented are all at zero water vapour concentration.

Following the derivation by Barbour *et al.* (2016) and Farquhar and Cernusak (2012) photosynthetic Δ^18^O discrimination was used to calculate *p*CO_2_ in the mesophyll cytosol, *C*_m_, with the assumption that *C*_m_ is equal to the *p*CO_2_ at the site of CO_2_–H_2_O exchange and assuming that cytosolic CO_2_ is in full isotopic equilibrium with local cytosolic water. This allowed *g*_m_ to be calculated from

$$g_{\text{m}}=A/\left(C_{\text{i}}-C_{\text{m}}\right)$$ (2)

$$C_{m}=C_{i}\left(\frac{\delta_{i}-a_{w}-\delta_{A}\left(1+a_{w}\right)}{\delta_{c}-a_{w}-\delta_{A}\left(1+a_{w}\right)}\right)$$ (3)

Equation 3 is the same as equation 21 of Barbour *et al.* (2016), and is a rearrangement of equation 18 of Farquhar and Cernusak (2012) using their notation. The oxygen isotope ratios are expressed relative to the standard, (VSMOW) (δx=(O 18/O 16)x(O 18/O 16)std−1). Intercellular *p*CO_2_ is denoted by *C*_i_, and *a*_w_ is the discrimination against C^16^O^18^O during liquid phase diffusion and dissolution (0.8‰).

The isotopic composition of CO_2_ being assimilated, δ_A_, is given by

$$\delta_{A}=\frac{\delta_{a}-\Delta^{18}O}{1+\Delta^{18}O},$$ (4)

where δ_a_ is the isotopic composition of ambient air (in our case δ_a_=δ_out_).

The oxygen isotope composition of CO_2_ in the intercellular airspaces, δ_i_, including ternary corrections proposed by Farquhar and Cernusak (2012), is given by

$$\delta_{i}=\frac{\delta_{io}+t\left[\delta_{A}\left(\frac{C_{a}}{C_{i}}+1\right)-\delta_{a}\frac{C_{a}}{C_{i}}\right]}{1+t}$$ (5)

where *C*_a_ is the *p*CO_2_ in the ambient air. The ternary correction factor, *t*, is given by

$$t=\frac{\left(1+\frac{a_{18bs}}{1000}\right)E}{2g_{ac}}$$ (6)

where *g*_ac_ is the total conductance to CO_2_, *E* the transpiration rate, and *a*_18bs_ is the weighted discrimination of C^16^O^18^O diffusion across the boundary layer and stomata in series given by:

$$a_{18bs}=\frac{\left(C_{a}-C_{s}\right)a_{18b}-\left(C_{s}-C_{i}\right)a_{18s}}{\left(C_{a}-C_{i}\right)}$$ (7)

where *C*_s_ is the *p*CO_2_ at the leaf surface and *a*_18s_ and *a*_18b_ are the discriminations against C^16^O^18^O through stomata and the boundary layer (8‰ and 5.8‰, respectively).

The isotopic composition of intercellular CO_2_ ignoring ternary corrections is given by

$$\delta_{io}=\delta_{A}\left(1-\frac{C_{a}}{C_{i}}\right)\left(1+a_{18bs}\right)-\frac{C_{a}}{C_{i}}\left(\delta_{a}-a_{18bs}\right)+a_{18bs}$$ (8)

To calculate *C*_m_, we assume that the isotopic composition of CO_2_ in the cytosol, δ_c_, is the isotopic composition of CO_2_ equilibrated with cytosolic water, δ_cw_, and

$$\delta_{\text{cw}}=\;\delta_{\text{w}}+\varepsilon_{\text{w}}$$ (9)

where δ_w_ is the stable oxygen isotope composition of water in the cytosol at the site of evaporation and ε_w_ is the isotopic equilibrium between CO_2_ and water (dependent on temperature *T*_K_ in K (Barbour *et al.*, 2016, and references therein).

$$\varepsilon_{w}\left(‰\right)=\frac{17604}{T_{K}}-17.93$$ (10)

### Calculation of the isotopic composition of water at the site of evaporation from the isotopic composition of transpired water

The isotopic composition of water at the site of evaporation, δ_w_, can be estimated from the Craig and Gordon model of evaporative enrichment (Craig and Gordon, 1965; Farquhar and Lloyd, 1993)

$$\delta_{w}=\delta_{t}+\varepsilon^{*}+\varepsilon_{k}+\frac{e_{a}}{e_{i}}\left(\delta_{wa}-\varepsilon_{k}-\delta_{t}\right)$$ (11)

where ε* is the equilibrium fractionation during evaporation, ε_k_ is the kinetic fractionation during vapour diffusion in air, δ_t_ is the oxygen isotopic composition of transpired water, *e*_a_/*e*_i_ is the ratio of ambient to intercellular vapour pressure, and δ_a_ is the isotopic composition of ambient air. ε* is dependent on temperature:

$$\varepsilon^{*}=2.644-3.206\left(\frac{10^{3}}{T_{K}}\right)+1.534\left(\frac{10^{6}}{T_{K}^{2}}\right)$$ (12)

ε_k_ is dependent on stomatal and boundary layer conductances and associated fractionation factors (Barbour *et al.*, 2016, and references therein):

$$\varepsilon_{k}=\frac{28g_{s}^{-1}+19g_{b}^{-1}}{g_{s}^{-1}+g_{b}^{-1}}$$ (13)

The isotopic composition of transpired water δ_t_ can be calculated from mass balance knowing the isotopic composition of the water entering δ_win_ and exiting δ_wout_ the leaf chamber (measured with the Picarro) and the water vapour concentration entering *w*_in_ and exiting *w*_out_ (measured with the LI-6400XT):

$$\delta_{t}=\left(\delta_{wout}\left(1-w_{in}\right)-\delta_{win}\frac{w_{in}}{w_{out}}\left(1-w_{out}\right)\right)\frac{w_{out}}{w_{out}-w_{in}}$$ (14)

### Calculation of the proportion of mesophyll cytosolic CO_2_ in equilibration with leaf water, θ

If *C*_m_ is known, it is possible to calculate the isotopic composition of cytosolic CO_2_ from measurements of Δ^18^O using equation 18 from Farquhar and Cernusak (2012):

$$\delta_{c}=\delta_{A}\left(1-\frac{C_{i}}{C_{m}}\right)\left(1+a_{w}\right)+\frac{C_{i}}{C_{m}}\left(\delta_{i}-a_{w}\right)+a_{w}.$$ (15)

This can then be compared with δ_cw_ (Equation 9), the isotopic composition of CO_2_ in equilibrium with water at the site of evaporation. We calculated mesophyll conductance, *g*_m_, in the *S. viridis* null plants assuming that δ_c_=δ_cw_ and then used this *g*_m_ to estimate *C*_m_ in the *S. viridis* transgenics to calculate the proportion of cytosolic CO_2_ in equilibration with leaf water, θ using equations developed by Cernusak *et al.* (2004)

$$\theta =\frac{\delta_{c}-\delta_{a}+a_{18}\left(1+\frac{C_{c}}{C_{a}}\right)}{\delta_{cw}-\delta_{a}+a_{18}\left(1+\frac{C_{c}}{C_{a}}\right)}$$ (16)

where *a*_18_ is the weighted discrimination of C^16^O^18^O diffusion across the boundary layer, stomata, and the liquid phase in series given by:

$$a_{18}=\frac{a_{b}\left(C_{a}-C_{s}\right)+a_{s}\left(C_{s}-C_{i}\right)+a_{w}\left(C_{i}-C_{m}\right)}{\left(C_{a}-C_{m}\right)}.$$ (17)

### Leaf anatomical measurements and estimation of *g*_m_ from anatomical measurements

Fully expanded leaves from 5-week-old T_2_ plants, null and line 1.1, were collected and cut into ~0.5×2mm pieces. Leaf slices were fixed in 2.5% (v/v) glutaraldehyde, 2% (v/v) paraformaldehyde, 0.1M phosphate buffer, and 0.01% (v/v) Tween-20 under vacuum for 20min, then replaced with buffer containing no Tween-20 and fixed overnight at 4 °C. Leaf pieces were washed in phosphate buffer and post-fixed in 1% (w/v) osmium tetroxide for 2h. Fixed leaf pieces were then dehydrated in an ethanol series (10, 30, 50, 70, 80, 95, 100%) followed by infiltration with LR white. Leaf sections were finally placed in moulds filled with resin and baked at 60 °C for 24h. Sections of 0.5 µm thickness were cut using glass knives on a Reichert ultramicrotome, stained with toluidine blue, and heat fixed to glass slides. Slides were viewed using a Zeiss Axioskop light microscope at ×400 magnification. Three images were taken from each slide for analysis, each containing a leaf cross-section in the same orientation and showing at least two vascular bundles. Fiji quantification software (Schindelin *et al.*, 2012) was used to select regions of interest. Mesophyll surface area exposed to intercellular airspace to leaf area ratio (*S*_m_) was calculated using Equation 18 where CCF is the curvature correction factor of 1.43 (Evans *et al.*, 1994).

$$S_{\text{m}}=\frac{\text{Length of mesophyll cells exposed to intercellular airspace}}{\text{Interveinal distance}}\times \text{CCF}$$ (18)

The values of *S*_m_ together with measurements of cell wall thickness and cytosol thickness were used to derive an estimate of *g*_m_ from anatomical parameters. The cell wall thickness (0.113±0.005 μm) was kindly estimated from transmission electron micrographs of *S. viridis* grown under similar conditions by Florence Danila (Danila *et al.*, 2016). Calculations followed equations 1–5 of von Caemmerer and Evans (2015) using the membrane permeability of Gutknecht for a lipid bilayer of 3.5×10^–3^ m s^−1^ since only the plasma membrane needs to be transversed for diffusion of CO_2_ from he intercellular airspace to mesophyll cytosol (Gutknecht *et al.*, 1977) and a cytosol thickness of 0.3 μm (von Caemmerer and Evans, 2015). These calculations give a *g*_m_ of 0.68mol m^−2^ s^−1^ bar^−1^.

### Statistical analysis

One-way ANOVAs with post-hoc Tukey test analyses were performed for all measurements of gas exchange and enzyme activities with *P*=0.05 using the IBM SPSS Statistics 22 package.

## Results

### Generation of transgenic S. viridis with reduced β-CA

In *S. viridis* we identified four β-CA genes: Si002140m.g (with one other isoform Si002148m), Si002669m.g, Si030616m.g (with two other isoforms Si030928m and Si030803m), and Si003882m.g. There is very low sequence identity between these β-CA genes, ~37% (Supplementary Fig. S2). Si003882m.g has been shown to be the major leaf β-CA (Christin *et al.*, 2013; John *et al.*, 2014).

Three independent transformation events resistant to hygromycin and with reduced CA activity were generated using two different approaches. First, one line (1.1) was generated through gene suppression upon transformation with the overexpression construct pSC110/*ZmCA2*. The coding sequences of *ZmCA2* and Si003882m.g show 87% identity (Supplementary Fig. S3). Most probably, expression of *ZmCA2* therefore caused suppression of the primary *S. viridis* β-CA gene, resulting in reduced CA activity in line 1.1. The second approach was to target Si003882m.g using the RNAi construct pSG/*CAa* which generated stably transformed lines from two different events (2.1 and 5.3). Plants were grown at high *p*CO_2_ for all experiments.

**Fig. 1.**
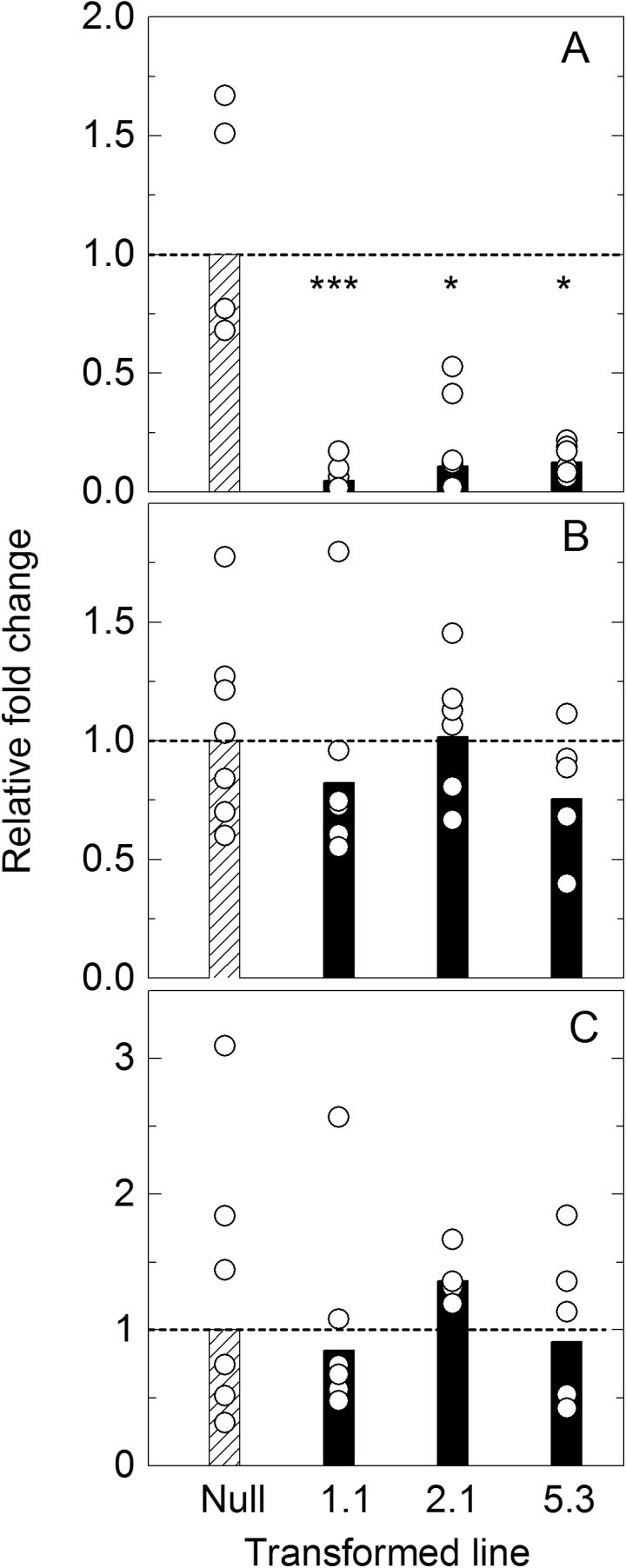
Expression level of β-CA transcripts. (A) Si003882m.g, (B) Si002140m.g, and (C) Si030616m.g in null control and CA transformed lines 1.1, 2.1, and 5.3 as measured by RT–qPCR and analysed by ΔΔCt. Fold change relative to the null transformant is shown; bars represent mean fold change, and circles show the data range of T_2_ plants (*n*=5–7 plants) from each transformation event measured in triplicate. The dotted line indicates average null fold change. Expression level of the major leaf β-CA transcript Si003882m.g (A) is significantly lower compared with the null control in all three transformed lines, calculated using one-way ANOVA.

To determine the specificity of the RNAi construct and check which β-CA was suppressed in line 1.1, RT–qPCR was performed against the β-CAs in *S. viridis*. Expression of the primary leaf β-CA Si003882m.g was significantly down-regulated, between 83% and 96%, in lines from all three transformation events (Fig. 1A). Transcript levels of Si030616m.g and Si002140m.g were unchanged relative to expression in the null plants (Fig. 1B, C) while Si002669m.g transcript was undetectable in all samples (data not shown). Therefore, expression of only the target β-CA gene was affected in the three transformed lines.

qPCR was used to estimate the number of insertions in the transgenic plants, based on the number of copies of the hygromycin phosphotransferase gene. Three T_2_ plants of the three lines were analysed and there were two, four, and more than four transgene insertions detected for plants of line 5.3, 2.1, and 1.1, respectively. The high copy number in the overexpressing line of 1.1 is the likely cause of the suppression of transcript accumulation.

### CA and photosynthetic enzyme activity and leaf anatomy

**Fig. 2.**
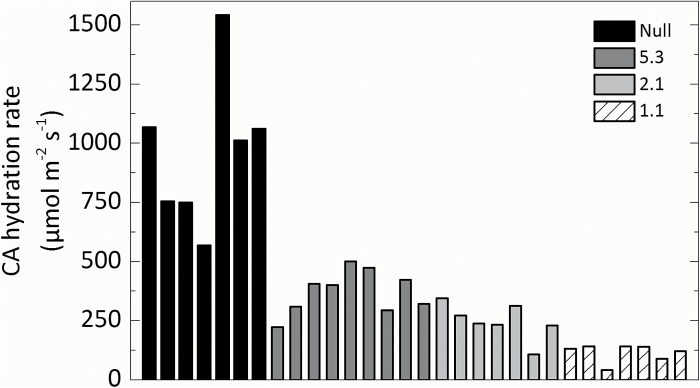
Range of CA hydration rates at mesophyll *p*CO_2_ (*C*_m_) measured using a membrane inlet mass spectrometer in the null control and T_2_ plants from lines 5.3, 2.1, and 1.1.

T_1_ progeny of the three independent transformation events showed a range of CA hydration rates as measured on the soluble leaf fraction on a membrane inlet mass spectrometer. Compared with the null control, lines 1.1, 2.1, and 5.3 had on average (*n*=7 T_2_ plants) an 87, 70, and 50% reduction of CA activity, respectively (Fig. 2). The CA hydration rate in the null plants was 934±92 µmol m^−2^ s^−1^ as calculated at a mesophyll *p*CO_2_ (*C*_m_) of 140 µbar (Equation 2).

The activities of the photosynthetic enzymes Rubisco, PEPC, and NADP-ME were unchanged in lines 5.3, 2.1, and 1.1 compared with the nulls (Table 1) and showed no correlation with CA hydration rates (one-way ANOVA and Tukey post-hoc analysis (SPSS statistics version 22; *P*=0.05).

No significant differences were observed for the surface area of mesophyll cells exposed to intercellular airspace per unit leaf area (*S*_m_) in embedded leaf sections of nulls (10.22±0.35 m^2^ m^−2^) and plants from line 1.1 (10.18±0.95 m^2^ m^−2^). These anatomical measurements were used to estimate an anatomical *g*_m_ of 0.68mol m^−2^ s^−1^ bar^−1^ (see the Materials and methods).

### CA activity and CO_2_ assimilation rates

**Fig. 3.**
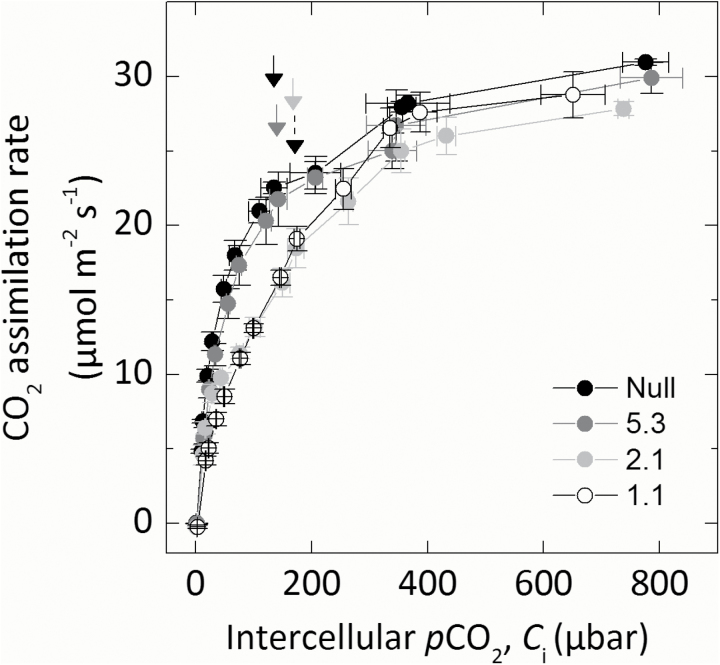
CO_2_ assimilation rate of transformed lines over a range of intercellular *p*CO_2_ (*C*_i_). Average of three T_2_ plants from each line. Plants were grown at 2% CO_2_, and the uppermost, fully expanded leaves of 5-week-old plants were measured using a LI-6400XT at 25 °C leaf temperature at an irradiance of 1500 µmol photons m^−2^ s^−1^. Arrows mark ambient *p*CO_2_ for each line; note that the dotted arrow is line 1.1.

**Fig. 4.**
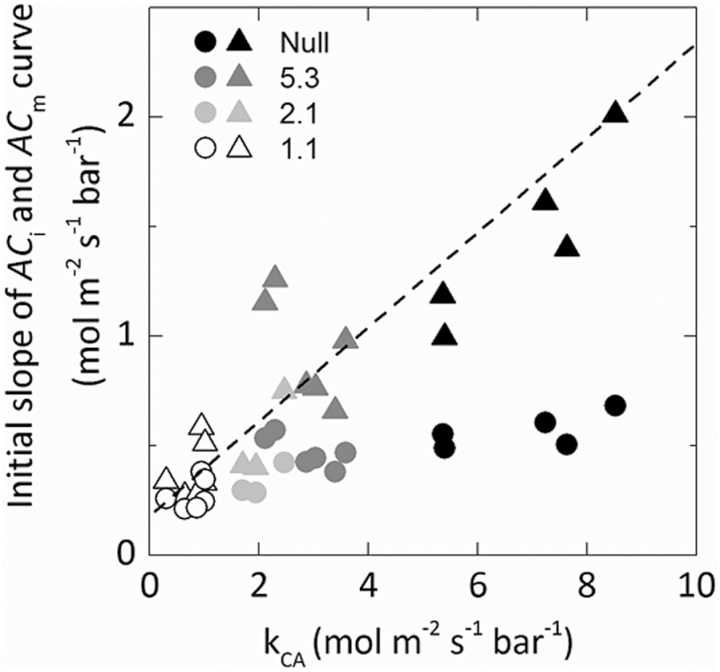
Relationship between the initial slope of the *AC*_m_ (triangles) or *AC*_i_ (circles) curves and the rate constant of CA hydration rates (*k*_CA_), *AC*_m_*R*^2^=0.846. Each point represents a measurement made on an individual leaf of a T_2_ plant.

**Fig. 5.**
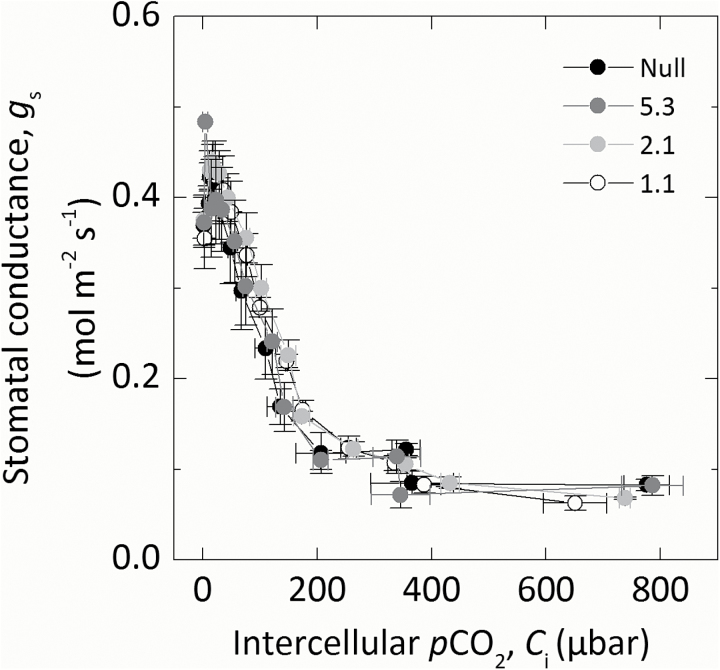
Stomatal conductance (*g*_s_) over a range of intercellular *p*CO_2_ (*C*_i_). Measurements were made concurrently with those in Fig. 4.

The response of CO_2_ assimilation rate (*A*) to increasing intercellular *p*CO_2_ (*C*_i_) was investigated to examine the effect of reduced CA activity on CO_2_ assimilation rates (Fig. 3). There were no statistical differences in the maximum rate of CO_2_ assimilation under ambient or high CO_2_ conditions between null control and progeny of transformant lines. At low *p*CO_2_, CO_2_ assimilation rates were reduced to varying degrees in the progeny of the transformed lines compared with the null control. Individuals of line 1.1 with the lowest CA hydration rate had the lowest initial slopes of the *AC*_i_ curves. The initial slopes of the *AC*_i_ and *AC*_m_ curve were plotted against the CA hydration rate constant (*k*_CA_; Fig. 4). Mesophyll cytosolic *p*CO_2_, *C*_m_, was calculated from Equation 2, using the average null *g*_m_ (0.9mol m^−2^ s^−1^ bar^−1^) since there was no difference in *S*_m_. A strong correlation between the initial slope from the *AC*_m_ curve and *k*_CA_ was observed, with the initial slope increasing as CA hydration rates increase (*R*^2^=0.845; Fig. 4). There was a curvilinear response between the initial slope of the *ACi* curves indicating other limitations. No difference in stomatal conductance (*g*_s_) was observed across a range of intercellular *p*CO_2_ between null controls and any of the transformed lines during the rapid measurements of CO_2_ responses (Fig. 5).

### Oxygen isotope discrimination measurements

Oxygen (∆^18^O) isotope discrimination and CO_2_ assimilation rates were measured in response to changes in *p*CO_2_ using a LI-6400XT coupled to a TDL trace gas analyser to measure C^18^O^16^O and a Cavity Ring-Down Spectrometer to measure the oxygen isotope composition of water vapour. Transformed plants with reduced CA hydration rates had lower ∆^18^O compared with the nulls, but only line 1.1 was significantly lower (Table 2).

**Fig. 6.**
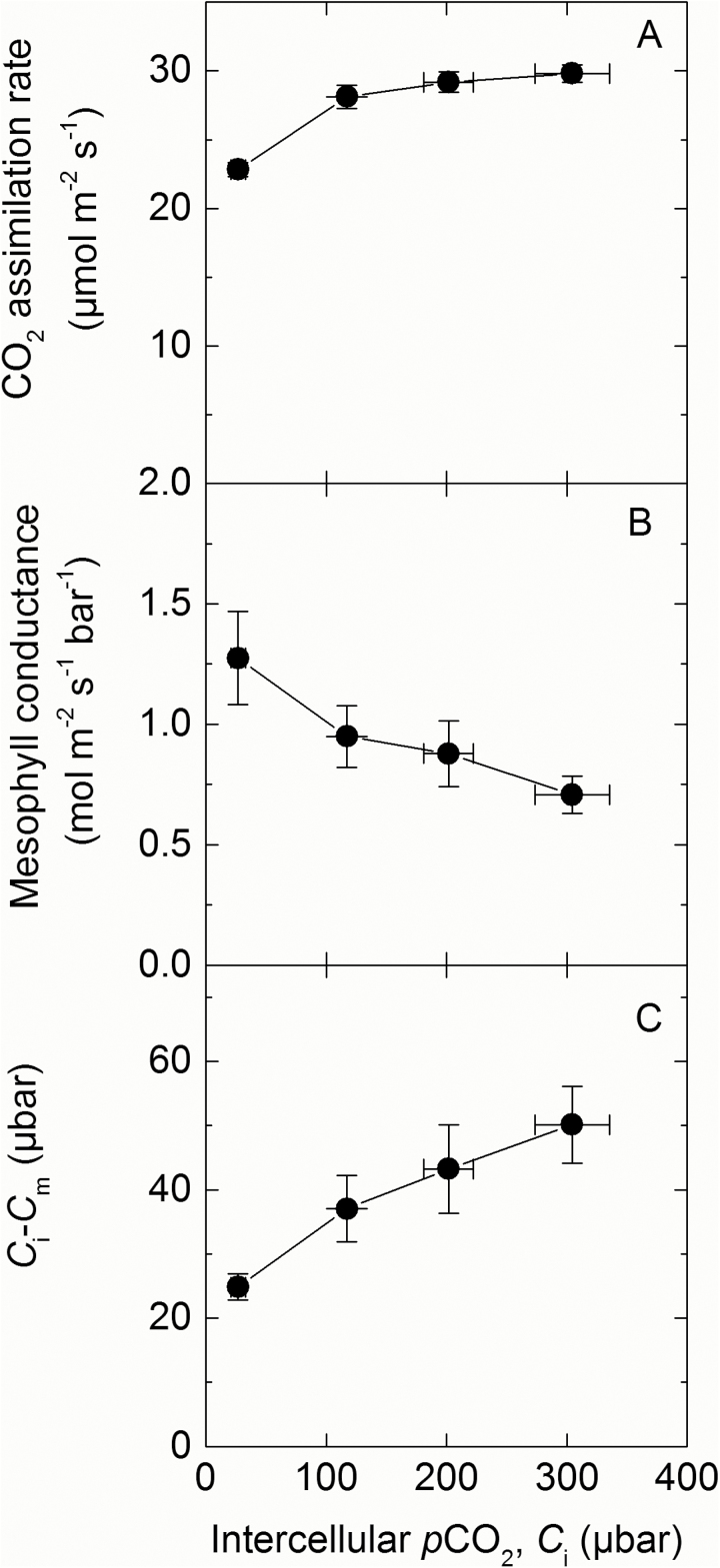
(A) CO_2_ assimilation rate, (B) mesophyll conductance (*g*_m_; Equation 2), and (C) *C*_i_–*C*_m_ over a range of intercellular *p*CO_2_ in null controls measured using a LI-6400XT coupled to a tunable diode laser. Plants were grown at 2% CO_2_ and the uppermost, fully expanded leaves of 5-week-old plants were measured at 25 °C leaf temperature, flow rate 200 µmol m^−2^ s^−1^, 2% O_2_ at an irradiance of 1500 µmol photons m^−2^ s^−1^.

In the null controls, measurements of ∆^18^O were used to estimate conductance of CO_2_ from the intercellular airspace to the sites of CO_2_ and H_2_O exchange in the cytosol (*g*_m_) with the assumption that CO_2_ was in full isotopic equilibrium with leaf water in the cytosol (Equation 2; Fig. 6). Although *g*_m_ appeared to increase with decreasing *C*_i_, there were no significant differences between *g*_m_ estimated at the different *C*_i_, and the average value was 0.94±0.06mol m^−2^ s^−1^ bar^−1^ (Fig. 6B). *C*_i_–*C*_m_ indicates the drawdown of CO_2_ from the intercellular airspace to the site of fixation, and for the null controls there is an increasing gradient of *p*CO_2_ as *C*_i_ increases (Fig. 6C).

**Fig. 7.**
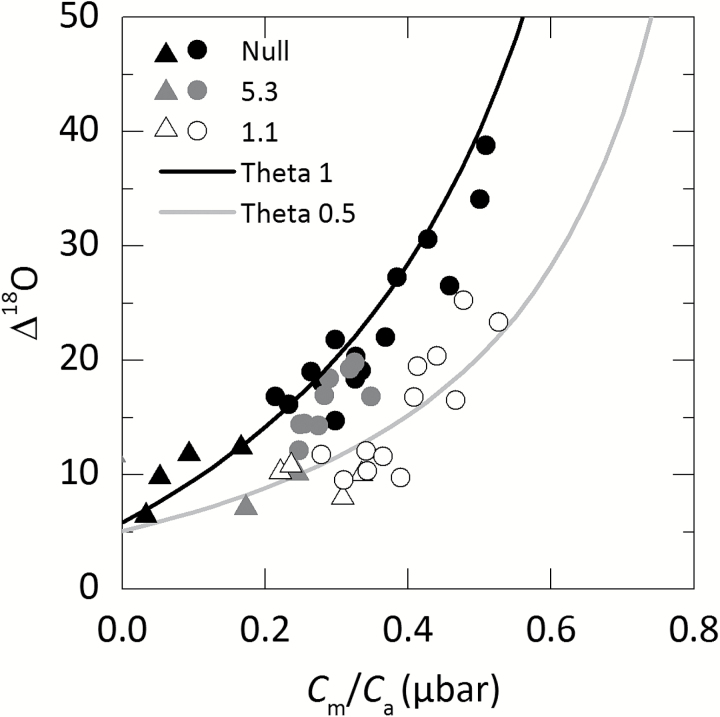
Oxygen isotope discrimination (Δ^18^O) as a function of the ratio of mesophyll *p*CO_2_ to ambient *p*CO_2_ (*C*_m_/*C*_a_) in null and lines 5.3 and 1.1. Each point represents a measurement made on an individual leaf of a T_2_ plant. Triangular symbols represent measurements made at low *p*CO_2_. Theoretical curves represent the scenario where cytosolic CO_2_ is at full isotopic equilibrium with cytosolic water (θ=1, black) or under partial equilibrium (θ=0.5, grey) of ^18^O in the leaf. The equations for the curves are given by ΔO 18=a18+CmCa−Cm(δc−δa)  and *a*_18_=5.85‰ and δ_c_–δ_a_=33‰ at full equilibration or *a*_18_=5.1‰ and δ_c_–δ_a_=15‰ (Farquhar and Lloyd, 1993).

**Fig. 8.**
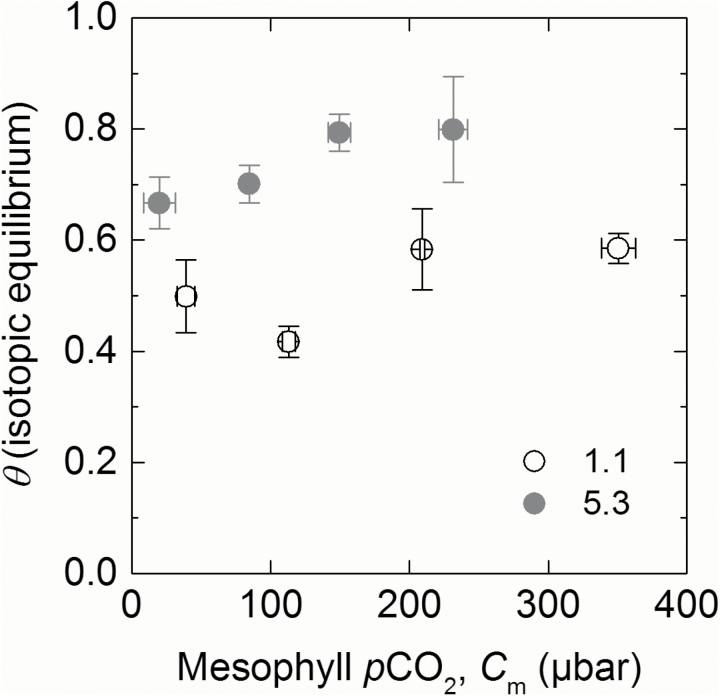
Average isotopic equilibrium (theta, θ) over a range of mesophyll *p*CO_2_ in two reduced CA lines 5.3 (grey) and 1.1 (white). Measured values of θ were determined from Δ^18^O using Equation 16. Each point represents the average measurement of three T_2_ plants.

∆^18^O at ambient *p*CO_2_ showed statistically significant differences between line 1.1 (with the lowest CA activity) and null plants (Table 2). When plotted against *C*_m_/*C*_a_, ∆^18^O measurements closely correspond to theoretical curves representing θ (Equation 16) under different scenarios either where cytosolic CO_2_ is at full isotopic equilibrium with the cytosolic water (null lines) or where there is only partial equilibrium (such as line 1.1; Fig. 7). Calculated values for line 5.3 which showed a 50% reduction in CA activity relative to the null controls fell in between these two theoretical lines. This is illustrated again with theta (θ) of lines 1.1 and 5.3 over a range of *C*_m_ (Fig. 8). When CO_2_ is at full isotopic equilibrium with the cytosolic water, θ would be 1, whereas in lines 1.1 and 5.3 (with reduced CA hydration rates relative to the null control) θ is <1. There was no CO_2_ dependence of θ over the range of *p*CO_2_ measured.

## Discussion

### *Setaria viridis* as a model species to study photosynthetic physiology in a C_4_ monocot

*Flaveria bidentis*, a readily transformable model C_4_ dicot, has been successfully used to study the regulation of C_4_ photosynthesis using antisense and RNAi technology (Furbank *et al.*, 1997; Matsuoka *et al.*, 2001; von Caemmerer *et al.*, 2004; Pengelly *et al.*, 2012). This work has been crucial in quantifying the rate-limiting steps in the C_4_ pathway by ‘titrating’ out levels of target enzymes by gene suppression and observing the effects on physiological characteristics of the resultant transgenics (Furbank *et al.*, 1997). There are, however, important differences between C_4_ dicots and the C_4_ monocots which make up the majority of agriculturally important C_4_ species. *Setaria viridis* has emerged as a new model grass to study C_4_ photosynthesis in crops and related bioenergy species. *Setaria viridis* is an appropriate biochemical model species for *Z. mays* and *S. bicolor* as all three use NADP-ME as the primary decarboxylation enzyme. We generated transgenic *S. viridis* plants with reduced CA activity to compare the effect with previous results obtained with *F. bidentis* and *Z. mays* (von Caemmerer *et al.*, 2004; Studer *et al.*, 2014) and to explore the effect that a reduction in CA activity has on the initial slope of the *AC*_i_ and *AC*_m_ curves. In these lines, only the major leaf isoform of β-CA was reduced (Fig. 1). The transgenic plants had a range of different CA activities (Fig. 2), but showed no changes in PEPC and Rubisco activity (Table 1) or anatomical parameters (Table 2), making these plants ideal for exploring the role of CA activity in *S. viridis*.

### Initial slope of *AC*_i_ curves in C_4_ plants

Models of C_4_ photosynthesis suggest that the initial slope of the *AC*_i_ curve is determined by three possible limitations: (i) the mesophyll conductance to CO_2_ diffusion from the intercellular airspace to the mesophyll cytosol (*g*_m_); (ii) the rate of CO_2_ hydration by CA; and (iii) the rate of PEP carboxylation (von Caemmerer, 2000). However, it is not readily known which is the major limitation in C_4_ species. Studies with PEPC mutants from the C_4_ dicot *Amaranthus edulis* indicate that PEPC activity may not be the major limitation as a 60% reduction in PEPC leads to only a 20% reduction in the CO_2_ assimilation rate at ambient *p*CO_2_ accompanied by a small reduction in initial slope for the *AC*_i_ curves (Dever *et al.*, 1992; Dever, 1997; Cousins *et al.*, 2007). This study with *S. viridis* confirms that substantial reductions in CA activity are possible before a reduction in steady-state CO_2_ assimilation rate and initial slope of the *AC*_i_ curve are observed. This is in accordance with previous observations in *F. bidentis* and *Z. mays* (von Caemmerer *et al.*, 2004; Studer *et al.*, 2014).

The Michaelis–Menten constant for CO_2_ for CA is >2mM (~5% CO_2_) which makes it appropriate to quantify CA activity by its first-order rate constant (Jenkins *et al.*, 1989; Hatch and Burnell, 1990) and simplifies species comparisons. In *S. viridis*, the lowest rate constant recorded was 0.8mol m^−2^ s^−1^ bar^−1^ compared with values of 0.1 for the *ca1ca2* double mutant in *Z. mays* and 0.47 for transgenic *F. bidentis* (von Caemmerer *et al.*, 2004; Studer *et al.*, 2014). With this low rate constant, *F. bidentis* had very low CO_2_ assimilation rates and the CO_2_ response curves did not saturate at high CO_2_. In contrast, for both *S. viridis* transgenics and *Z. mays* mutants, CO_2_ assimilation rates were only slightly less than in the controls, suggesting that *S. viridis* is more similar to *Z. mays* in its CA requirements. This suggests that these two monocot species can make better use of leaf CA activity or that *in vivo* CA activity is greater than that estimated *in vitro*.

### Mesophyll conductance and the initial slope of *AC*_m_ curves

Next, we used recently established techniques that utilize ^18^O discrimination measurements to quantify *g*_m_ in our null controls (Fig. 6B; Barbour *et al.*, 2016). This estimates the diffusion of CO_2_ from the intercellular airspace through the cell wall, plasma membrane, and cytosol to the sites of CA activity. At ambient *p*CO_2_, the *g*_m_ observed for the null plants were similar to those reported by Barbour *et al.* (2016). A key assumption for the calculation of *g*_m_ is that CA activity is not limiting and that CO_2_ is in isotopic equilibrium with HCO_3_^−^; consequently *g*_m_ was not measured in the transgenic lines with reduced CA activity. In C_3_ species, *g*_m_ (in this instance from the intercellular airspace to the chloroplast stroma) has been shown to be proportional to the chloroplast surface area appressing the intercellular airspace per unit leaf area (Evans *et al.*, 1994). Evans and von Caemmerer (1996) hypothesized that in C_4_ species *g*_m_ may correlate with the mesophyll surface area exposed to intercellular airspace per unit leaf area (*S*_m_). Since *S*_m_ was similar between the nulls and line 1.1 plants, we assumed that *g*_m_ may also be similar between the plants. In C_3_ species, *g*_m_ has been shown to, in some instances, increase with decreasing *p*CO_2_ (Flexas *et al.*, 2007; Tazoe *et al.*, 2011; Alonso-Cantabrana and von Caemmerer, 2016). These changes to *g*_m_ which may be important in regulating and maintaining photosynthesis were also observed here in the *S. viridis* null plants, with *g*_m_ increasing slightly at low *p*CO_2_. However, because the differences in *g*_m_ at different *p*CO_2_ were not significant, we used the average *g*_m_ estimated for the null plants to calculate mesophyll cytosolic *p*CO_2_ (*C*_m_) in the transgenics.

As shown in Fig. 4, a strong almost linear relationship was found between *AC*_m_ and *k*_CA_, whereas a saturating relationship was observed with *AC*_i_. This indicates that the CO_2_ assimilation rate is limited by cytosolic CA activity, with the relationship becoming clearer after accounting for *g*_m_. It is tempting to speculate that the differences between the two monocot species and *F. bidentis* relate to differences in limitations imposed by *g*_m_ which affects cytosolic *p*CO_2_ and hence *in vivo* CA activity, but this is not borne out by comparative measurements of *g*_m_ made by Barbour *et al.* (2016). CA activity increases with increasing pH, so variation in cytosolic pH can also contribute to variations in *in vivo* CA activity; however, these effects are not large (Jenkins *et al.*, 1989). The interaction of β-CA and a CO_2_-permeable aquaporin in *Arabidopsis thaliana* has indicated that CA can be localized near the plasma membrane rather than dispersed throughout the mesophyll cytosol (Wang *et al.*, 2016). This may also impact on CA activity and result in another difference between the C_4_ species. Other possibilities pertain to differences in anatomical characteristics of leaves. Both CA and PEPC are cytosolic enzymes, and differences in *S*_m_ may affect the efficiency with which CA is used. Our results suggest that increasing *g*_m_ may be an important way to increase the CO_2_ assimilation rate at low intercellular *p*CO_2_, a scenario that may, for example, occur under drought.

### Oxygen isotope discrimination and the CO_2_ dependence of isotopic equilibrium

As had previously been observed, Δ^18^O decreased with reductions in CA activity as CA facilitates the exchange of O_2_ between cytosolic water and CO_2_ (Fig. 7; Williams *et al.*, 1996; Cousins *et al.*, 2006). Previous reports, which have estimated the proportion of cytosolic CO_2_ in equilibrium with leaf water (θ) in C_4_ species, have generally assumed a relatively large *g*_m_ value and this then led to lower estimates of θ (Cousins *et al.*, 2006, 2008). Here we assumed that in the *S. viridis* null plants there is sufficient CA for isotopic equilibrium to be reached, as discussed by Barbour *et al.* (2016). For comparison, we also estimated *g*_m_ from anatomical estimates of *S*_m_, and cell wall and cytosolic thickness following calculations outlined by von Caemmerer and Evans (2015). This gives a *g*_m_ value of 0.68mol m^−2^ s^−1^ bar^−1^ which is less than the value of 0.9mol m^−2^ s^−1^ bar^−1^ calculated from Δ^18^O measurements and highlights the anatomical constraints for CO_2_ diffusion dictated by the photosynthetic pathway in leaves of C_4_ plants (von Caemmerer *et al.*, 2007).

Reduction in CA activity led to significant reductions in θ but it is interesting to note that θ did not vary significantly with *p*CO_2_. This is explained by the fact that CA activity increases linearly with *p*CO_2_ so that although there is more CO_2_ that needs to equilibrate with leaf water, there is also proportionally more CA activity. The fact that neither transgenic line showed a CO_2_ dependence suggests that the decrease in the ratio of CA hydrations to PEP carboxylations is not affecting the isotopic equilibration of CO_2_ with leaf water. These results have important implications for the interpretation of the ^18^O signature of atmosopheric CO_2_ (Yakir and Sternberg, 2000; Gillon and Yakir, 2001; Wingate *et al.*, 2009).

### Reduction of CA in *S. viridis* does not alter the stomatal reponse to CO_2_

The CO_2_ regulation of stomatal conductance remains an open question (Engineer *et al.*, 2016). It has been previously shown that in the *ca1/ca4* double mutant of *A. thaliana*, the degree of stomatal closure in response to increasing *p*CO_2_ was reduced (Hu *et al.*, 2010; Wang *et al.*, 2016). It is clear that CA is part of a complex signal transduction network. However, nothing is currently known about the role of CA in stomatal CO_2_ responses in C_4_ species. In our study, where only one β-CA isoform was reduced, we found no change in the response of stomatal conductance to CO_2_. The *S. viridis* β-CA reduced here (Si003882m.g) has low sequence identity (<50%) to all of the Arabidopsis β-CAs, but we would predict that multiple reductions in β-CA isoforms would be required to observe a similar stomatal phenotype in *S. viridis*.

### Conclusion

Under current atmospheric conditions, CA activity was not rate limiting for C_4_ photosynthesis in *S. viridis.* At lower *C*_i_, which may, for example, occur under conditions of drought, our results suggest that *g*_m_ may pose a greater limitation than CA activity. However, it is important to investigate the role of CA on C_4_ photosynthesis under a range of environmental conditions such as high temperatures which have recently been suggested to deactivate CA activity in *S. viridis* (Boyd *et al.*, 2015). Here we have shown that *S. viridis* is a useful model monocot C_4_ species that lends itself to molecular manipulation of the C_4_ photosynthetic pathway.

## Supplementary Data

Supplementary data are available at *JXB* online.

Table S1. Primers used in this study

Figure S1. CA hydration rates at mesophyll *p*CO_2_ in the T_1_ plants.

Figure S2. Very low sequence identity (~37%) between the four main *S. viridis* β-CAs.

Figure S3. High sequence identity (87%) of Si003882m.g to the *ZmCA2* (GRMZM2G348512).

Figure S4. CO_2_ assimilation rate of the TDL experiment.

Figure S5. Standard error of δ^18^O in the reference gas of repeated measurements with the TGA200A.

## Supplementary Material

Supplementary_Table_S1_Figures_S1_S5
