# Supplementary material for: Effects of reduced carbonic anhydrase activity on CO2 assimilation rates in Setaria viridis: a transgenic analysis
Source: J Exp Bot. 2016 Oct 4;68(2):299–310. doi: 10.1093/jxb/erw357 (PMC5853810; doi:10.1093/jxb/erw357)
Supplement: Supplementary_Table_S1_Figures_S1_S5 [file erw357_suppl_supplementary_table_s1_figures_s1_s5.pdf]

Supplementary Table 1: Primers used in this study.

Gene	Description	Sequence 5' - 3'	Amplicon Size (bp)	Use
Si016746m	Ubiquitin	F: GATCTCCGCCCCAGCAAGAT<br><br>R: ATGCCCTCCTTGTCCTGGAT	124	RT-qPCR housekeeping
Si022040m	Elongation factor 1a	F: GCTGCAACAAGATGGATGCC<br><br>R: CCAGAGATTGGGACGAAGGC	132	RT-qPCR housekeeping
Si035709m	Beta tubulin	F: CTAAAGCTCGCCACCCCTAC<br><br>R: GTCGGAGTTGAGCTGACCAG	104	RT-qPCR housekeeping
Si030616m	β CA	F: AGATGATTCAACCTCTGGAAGCT<br>R: TTGCACTGCATTTCAAAACTCA	110	RT-qPCR
Si002140m	β CA	F: AGGCCGACAAGTTCCACTTC<br>R: CATTGGTCCTCGAAAGCAGC	102	RT-qPCR
Si002669m	β CA	F: GGCTGGGTTCAGGACGTTTA<br>R: AGAGTCAGAGCACGCAAACA	112	RT-qPCR
Si003882m	β CA	F: CATAAATTCCCCCGCCTCGT<br>R: CTCTTCAAGCGCTCGACGG	101	RT-qPCR
Si003882m	β CA	F: GAGGGCCAGGCACCCAGGTA<br>R: GACGGCGTACTCGATGGCGG	177	pSG/CAa insert
	Hygromycin phosphotransferase	F: TGGCGTGATTTCATATGCGC<br>R: CGTCAACCAAGCTCTGATAG	420	Genotyping
GRMZM2G348512	ZmCA2	F:<br>CACCATGGACGACCCCGTCGAGCGCTTGAAGGAC<br>R:<br>TCAAGACCAGCCGCTCGCATCTTTCCAAGACGATG<br>GCTGCTTATTGTCC	625	pSC110/ZmCA2 insert

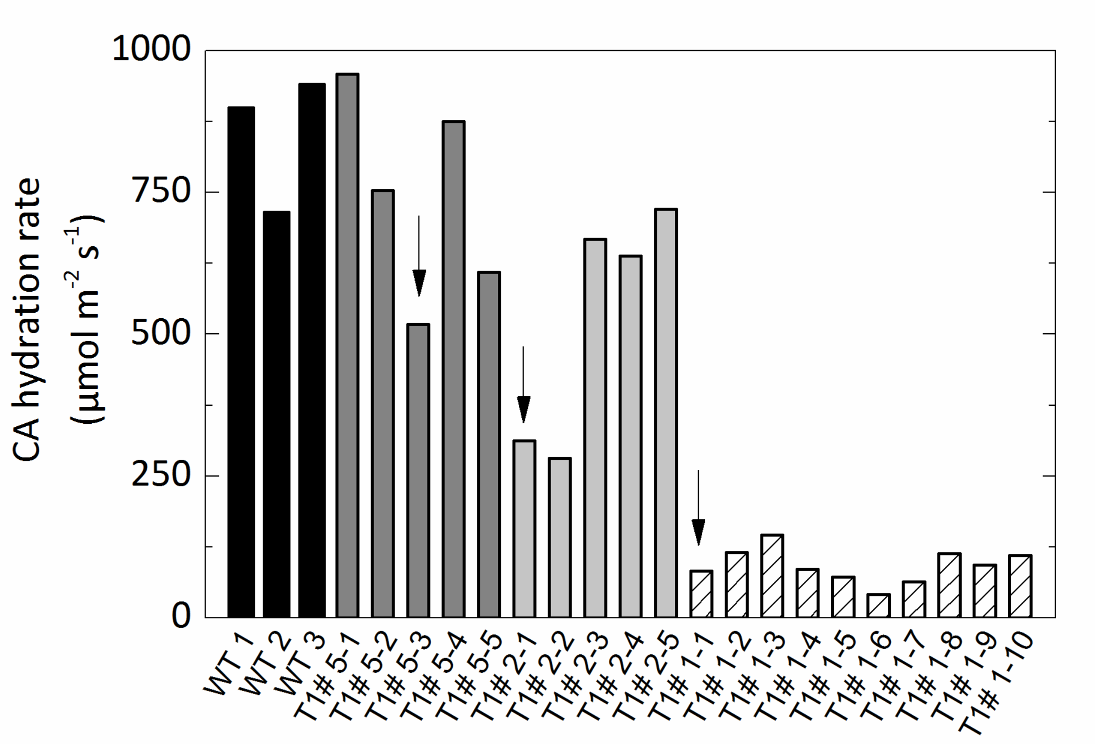

Supplementary Figure 1: CA hydration rates at mesophyll *p*CO_2 in the T_1 plants from three transformation events. T_0 lines #5 and #2 were generated using the RNAi vector pSG/CAa. T_0 line #1 was generated by gene suppression with pSC110/*ZmCA2*. Arrows indicate the T_1 plants with low CA hydration rates whose progeny were then used for future studies and are labelled T1#5-3,T1#2-1 and T1#1-1.

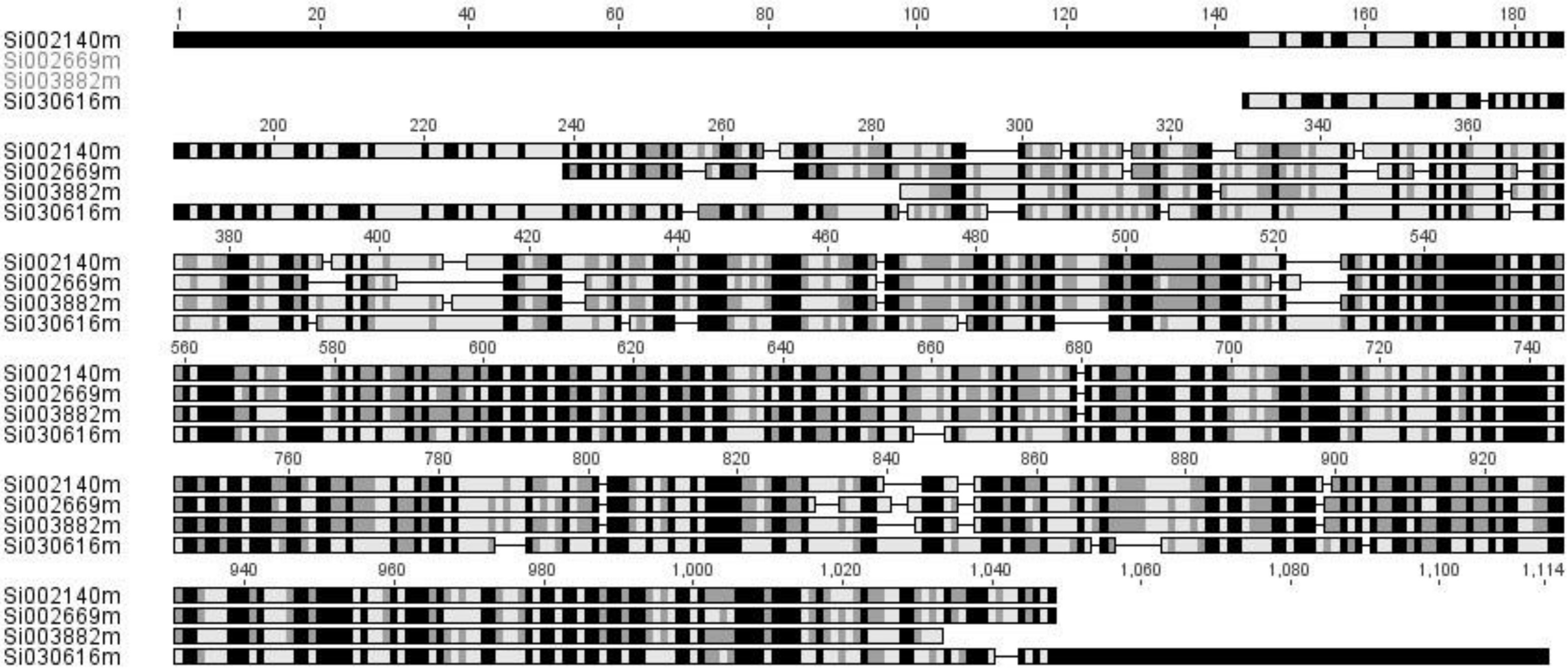

Supplementary Figure 2: Very low DNA sequence identity (~37%) between the four main Setaria β-CAs using Clustal-W alignment.

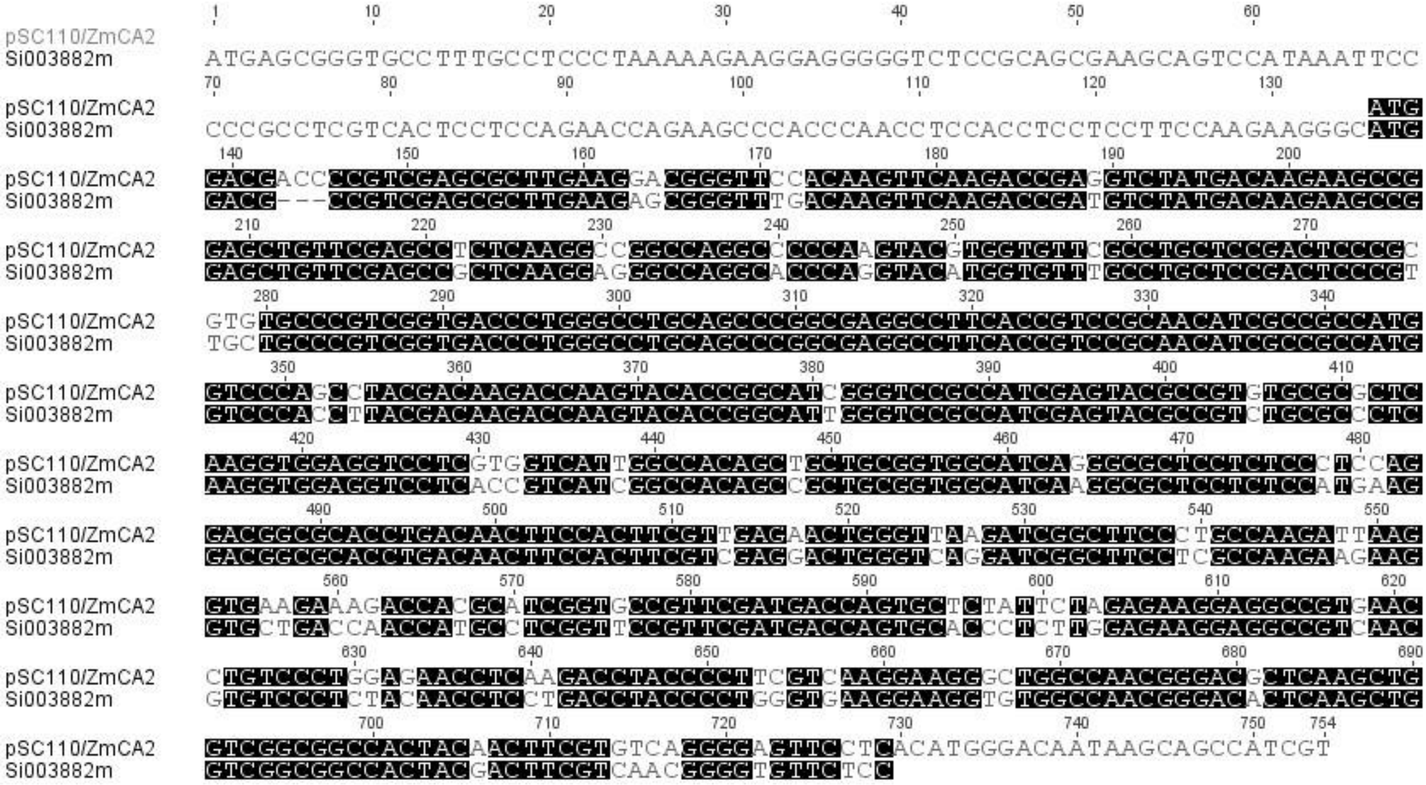

Supplementary Figure 3: High sequence identity (87%) of Si003882 to the *ZmCA2* (GRMZM2G348512) used for transformation in overexpression construct pSC110/ZmCA2 using Clustal-W alignment.

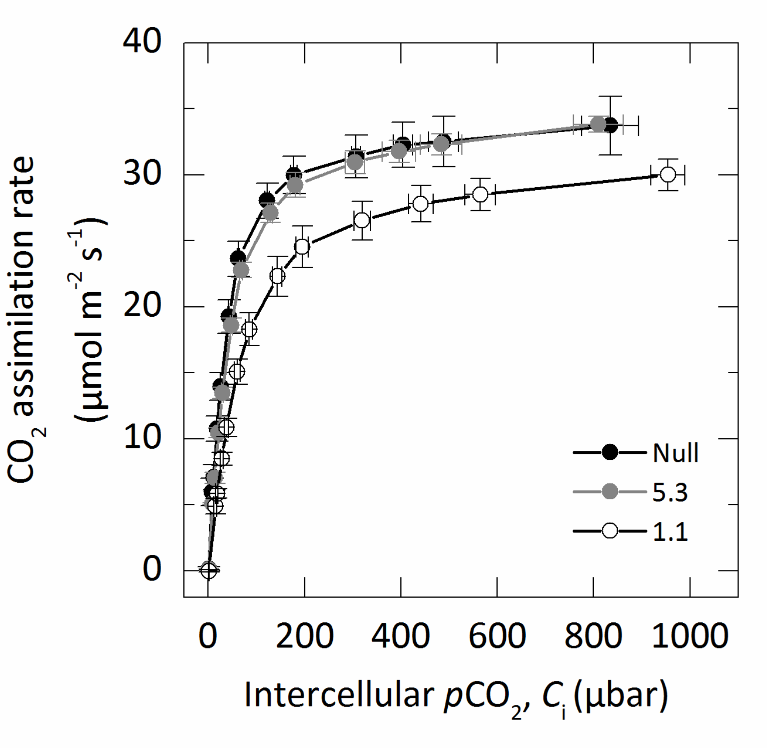

Supplementary Figure 4: CO_2 assimilation rate of transformed lines over a range of intercellular pCO_2 (C_i). Average of three T_2 plants of lines 5.3 and 1.1 were grown for TDL measurements. Plants were grown at 2 % CO_2 and the uppermost, fully expanded leaf of 5 week old plants were measured using a Li6400 at 25 °C leaf temperature at an irradiance of 1500 µmol photons m^{-2} s^{-1}.

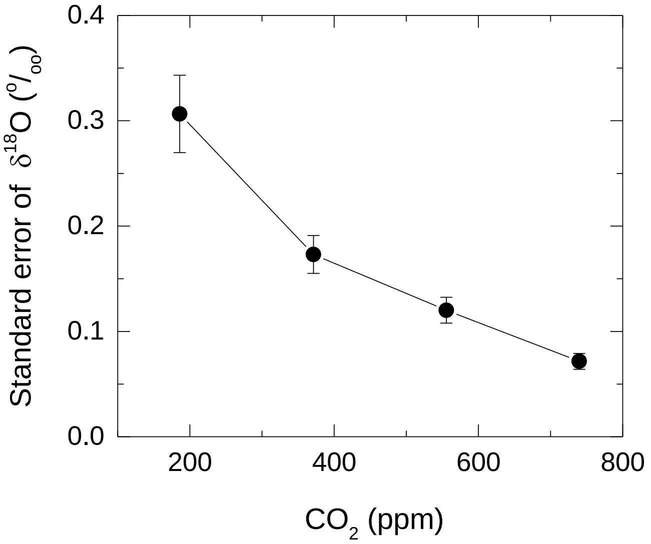

Supplementary Figure 5: Standard error of $\delta^{18}O$ in the reference gas of repeated measurements on the TGA200A. Each point represents the average ± s.e. of standard errors calculated from eight different experiments.
